# Zika Virus IgG in Infants with Microcephaly, Guinea-Bissau, 2016

**DOI:** 10.3201/eid2405.180153

**Published:** 2018-05

**Authors:** Maiken Worsøe Rosenstierne, Frederik Schaltz-Buchholzer, Fernanda Bruzadelli, Asson Có, Placido Cardoso, Charlotte Sværke Jørgensen, Johan Michiels, Leo Heyndrickx, Kevin K. Ariën, Thea Kølsen Fischer, Anders Fomsgaard

**Affiliations:** Statens Serum Institut, Copenhagen, Denmark (M.W. Rosenstierne, F. Schaltz-Buchholzer, C.S. Jørgensen, T.K. Fischer, A. Fomsgaard);; Bandim Health Project, Bissau, Guinea-Bissau (F. Schaltz-Buchholzer);; Field Epidemiology Training Program, Bissau (F. Bruzadelli, A. Có);; Instituto Nacional de Saúde Pública, Bissau (P. Cardoso);; Institute of Tropical Medicine Antwerp, Antwerp, Belgium (J. Michiels, L. Heyndrickx, K.K. Ariën);; University of Antwerp, Antwerp (K.K. Ariën);; University of Southern Denmark, Odense, Denmark (A. Fomsgaard)

**Keywords:** Zika virus, TORCH, microcephaly, congenital CMV, viruses, vector-borne infections, *Toxoplasma gondii*, *Treponema pallidum*, varicella-zoster virus, parvovirus B19, rubella virus, cytomegalovirus, herpes simplex virus, Guinea-Bissau, infants, CMV, congenital infections, birth defects

## Abstract

We analyzed blood samples from infants born with microcephaly and their mothers in Guinea-Bissau in 2016 for pathogens associated with birth defects. No Zika virus RNA was detected, but Zika virus IgG was highly prevalent. We recommend implementing pathogen screening of infants with congenital defects in Guinea-Bissau.

In 2016, the health authorities in Guinea-Bissau reported 4 cases of Zika virus infection and 5 cases of microcephaly (*1*) to the World Health Organization. The Zika virus strain detected in Guinea-Bissau was the African strain (*1*) originally detected in Africa in 1947 and in Portuguese Guinea (now Guinea-Bissau) during 1964–1965 (*2*). As of March 2018, the Asian strain, which has spread throughout the Americas and Cape Verde (*2*) and is linked to microcephaly and other congenital abnormalities, has not been reported in Guinea-Bissau (*3*), and the African Zika virus strain has not been linked with microcephaly.

We report an in-depth investigation of pathogens commonly associated with birth defects in 15 infants born with microcephaly in Guinea-Bissau in 2016. Field epidemiologists identified cases of microcephaly through reports from health center personnel across the country and surveillance at Hospital Nacional Simão Mendes in Bissau, Guinea-Bissau (which has 6,000 births/y). Most cases were found in the northern and eastern regions (Gabú, Bafatá, and Oio) of Guinea-Bissau (Technical Appendix Tables 1, 2). Blood samples were collected from the mothers (median age 22 years, range 15–31 years) and infants (median age 5 months, range 1 day–9 months) and sent to Statens Serum Institut (Copenhagen, Denmark) for analysis. Three infants died before sampling, and 1 sample was lost during transport; hence, we analyzed blood samples from 11 of the 15 infants with microcephaly. For comparison, we also analyzed blood samples from 10 mothers (from Tantam Cossé, Bafatá region) of infants born without microcephaly (M.W. Rosenstierne, unpub. data). We assayed for Zika virus and TORCH pathogens (*Toxoplasma gondii*, other [*Treponema pallidum*, varicella-zoster virus, parvovirus B19], rubella virus, cytomegalovirus [CMV], and herpes simplex virus) (Technical Appendix Tables 1, 2) because these pathogens are most commonly associated with congenital anomalies (*4*,*5*).

Zika virus IgG immunofluorescence assay and Zika virus neutralization test (*6*,*7*) results revealed that 14 (93%) of the 15 mothers of infants with microcephaly had Zika virus neutralizing antibodies (NAbs) (Technical Appendix Tables 1, 2) versus 5 (50%) of the 10 mothers of healthy infants (data not shown). We tested blood samples from the 11 infants with microcephaly for Zika virus NAbs, and all were positive (presumably maternal antibodies) (Technical Appendix Tables 1, 2). We did not perform this assay with samples from the healthy infants. No samples were positive for Zika virus RNA or IgM or had cross-neutralizing antibodies to dengue virus. Thus, the Zika virus seroprevalence among Guinea-Bissau women was surprisingly high and significantly higher in the mothers of infants with birth defects (p = 0.02 by Fisher exact test). However, timing of the Zika virus infection and strain could not be determined.

Because of sample volume limitations, we tested only 10 of 15 mothers for TORCH antibodies and all 11 infants with birth defects and available blood samples for TORCH pathogen nucleic acids (Technical Appendix Tables 1, 2). Four infant blood samples were positive for CMV DNA and IgG but only 2 were positive for CMV IgM (Technical Appendix Tables 1, 2). Two of these infants’ mothers were CMV IgG positive (the other 2 were not tested), and 1 mother tested positive for CMV IgM. Because sampling of infants was mainly performed 5 months postpartum rather than during the first 2–3 weeks postpartum (*5*,*8*), determining whether the CMV infections were congenital or acquired perinatally or postnatally (e.g., through breast milk) was not possible.

The mother whose infant died 5 days after birth was positive for *Toxoplasma* IgG (Technical Appendix Tables 1, 2). However, samples from this child were not collected for analysis, so we could not determine whether the infant died of severe congenital toxoplasmosis. As expected, almost all mothers were positive for antibodies against parvovirus (70%), varicella-zoster virus (90%), rubella virus (90%), CMV (90%), and herpes simplex virus (100%).

Although we found a high prevalence of Zika virus NAbs and TORCH antibodies in mothers and infants, the late sampling of infants and lack of Zika virus RNA–positive samples precludes determination of the cause of microcephaly in these infants. On the basis of our findings, we propose implementing prospective surveillance in Guinea-Bissau for infants with easily identifiable congenital abnormalities, such as microcephaly (i.e., head circumference 2 standard deviations below average for age and sex) (*9*), microphthalmia, and hearing loss, and screening these infants for Zika virus and TORCH by using blood, saliva, and urine samples collected immediately or within the first 2–3 weeks after birth. The low prevalence (0.6%) of microcephaly reported in 2015 (*10*) makes this suggestion feasible in resource-poor countries. If the Asian Zika virus strain is detected in Guinea-Bissau, screening of pregnant women during their first trimester should also be implemented. However, the 2-step surveillance and screening model can be applied in countries without reported detection of the Asian Zika virus strain.

Technical Appendix Epidemiologic characteristics and diagnostics test results of infants with microcephaly and their mothers, Guinea-Bissau, 2016.
