# Supplementary material for: Zika Virus IgG in Infants with Microcephaly, Guinea-Bissau, 2016
Source: Emerg Infect Dis. 2018 May;24(5):948–50. doi: 10.3201/eid2405.180153 (PMC5938792; doi:10.3201/eid2405.180153)
Supplement: Technical Appendix — Epidemiologic characteristics and diagnostics test results of infants with microcephaly and their mothers, Guinea-Bissau, 2016. [file 18-0153-Techapp-s1.pdf]

Article DOI: https://doi.org/10.3201/eid2405.180153

Zika Virus IgG in Infants with Microcephaly, Guinea-Bissau, 2016

Technical Appendix

Technical Appendix Table 1. Epidemiologic characteristics and diagnostics test results of infants with microcephaly and their mothers, cases A-G, Guinea-Bissau, 2016*

Category	Case													
	A		B		C		D		E		F		G	
Patient	Infant†	Mother	Infant	Mother	Infant†	Mother	Infant†	Mother	Infant	Mother	Infant	Mother	Infant	Mother
Age	1 m‡	18 y	4 m	24 y	5 d‡	18 y	9 m	30 y	3 m	20 y	5 m	30 y	5 m	22 y
Image of infant	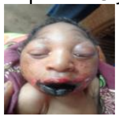		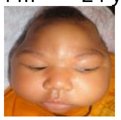		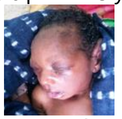		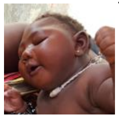		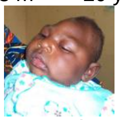		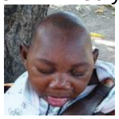		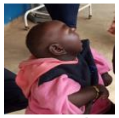	
Region	Bafatá		Bafatá		Bafatá		Oio		Gabú		Gabú		Bafatá	
Village	Sta Gaulo		Tantam Cossé		Contubel		Nhacra		Buruntuma		Duma		Tantam Cossé	
Symptoms of mother during pregnancy	Periodic headache		Weakness		None		None		Periodic headache		Fever		Fever, headache, weakness, stomach pain	
Month of birth	August		September		August		June		May		June		May	
Birthweight	Not reported		Not reported		Not reported		2,900 g		2,800 g		Not reported		Not reported	
Weight at sampling	2,200 g		3,550 g		Not reported		Not reported		Not reported		Not reported		6,200 g	
Infant sex	M		F		F		F		F		M		M	
Infant head circumference (*1*) at birth	25 cm		26 cm		25 cm		Not reported		Not reported		Not reported		Not reported	
Girls: reference range (31.7–36.1 cm)														
Boys: reference range (32.1–36.9 cm)														
Infant head circumference at sampling	Not reported		Not reported		Not reported		31.5 cm		Not reported		Not reported		35 cm	
Girls: reference range (39.0–43.9 cm)														
Boys: reference range (40.3–44.8 cm)														
Clinical symptoms	Microcephaly		Microcephaly		Microcephaly		Microcephaly		Microcephaly		Microcephaly		Microcephaly	
Pathogen, diagnostic test														
Zika virus														
*RT-q*PCR§	NA	–	–	–	NA	–	NA	–	–	–	–	–	–	–
IgM¶	NA	–	–	–	NA	–	NA	–	–	–	–	–	–	–
IgG¶	NA	+	+	+	NA	–	NA	+	+	+	+	+	+	–
Nab#	NA	>640	200	>640	NA	>640	NA	480	240	400	>640	538	80	>640
Dengue virus														
IgM¶	NA	–	–	–	NA	–	NA	–	–	–	–	–	–	–
IgG¶	NA	+	–	+	NA	+	NA	–	+	+	–	–	–	–
Nab#	NA	NA	–	NA	NA	NA	NA	NA	53	NA	NA	NA	–	NA
Chikungunya virus														

Category	Case													
	A		B		C		D		E		F		G	
Patient	Infant†	Mother	Infant	Mother	Infant†	Mother	Infant†	Mother	Infant	Mother	Infant	Mother	Infant	Mother
IgM¶	NA	–	–	–	NA	–	NA	–	–	–	–	–	–	–
IgG¶	NA	+	+	+	NA	+	NA	–	+	+	–	–	–	–
Toxoplasma gondii														
*IgM***		NA		–		–		–		–		NA		–
*IgG***				–		230		–		–				–
Treponema pallidum														
IgG††		NA		–		–		–		–		NA		–
Parvovirus														
*q*PCR‡‡	NA		–		NA		NA		–		–		–	
IgG¶¶		NA		0.74		0.90		0.64		0.90		NA		0.89
Varicella-zoster virus														
*q*PCR‡‡	NA		–		NA		NA		–		–		–	
IgG††		NA		INC		800		800		800		NA		200
Rubella virus														
*q*PCR‡‡	NA		–		NA		NA		–		–		–	
IgG§§		NA		80		102		87		160		NA		INC
Cytomegalovirus														
*q*PCR‡‡	NA		–		NA		NA		–		–		–	
IgM††		NA		NA		NA		NA		NA		NA		NA
IgG††		NA		>640		40		-		80		NA		5
Herpes simplex virus														
*q*PCR‡‡	NA		–		NA		NA		–		–		–	
IgG††		NA		1.98		1.85		2.23		1.82		NA		1.65

*INC, inconclusive; NA, not analyzed; Nab, neutralizing antibody; RT-qPCR, reverse transcription quantitative PCR; qPCR, quantitative PCR.
†Samples not send to Statens Serum Institut (Copenhagen, Denmark) for analysis.
‡Deceased.
§In-house PCR modified from Faye et al (*2*).
¶Arbovirus Fever Mosaic 2 (Zika virus, chikungunya virus, dengue virus) (IgG/IgM) (Euroimmun AG,Luebeck, Germany).
#Neutralization assay with African strain MR766. Positive antibody titers are shown.
**VIDAS TOXO IgG II (bioMérieux, Marcy l'Etoile, France). Positive antibody titers are shown.
††In-house ELISA. Positive antibody titers are shown.
‡‡In-house qPCR. Positive Cq values are shown.
§§ Enzygnost anti-Rubella-Virus IgG (Siemens Healthineers, Erlangen, Germany). Positive antibody titers are shown.
¶¶LIAISON Biotrin Parvovirus B19 IgG (DiaSorin, Saluggia, Italy). Positive antibody titers are shown.

Technical Appendix Table 2. Epidemiologic characteristics and diagnostics test results of infants with microcephaly and their mothers, cases H-O, Guinea-Bissau, 2016*

Category	Case H		Case I		Case J		Case K		Case L		Case M		Case N		Case O	
Patient	Infant	Mother	Infant	Mother	Infant	Mother	Infant	Mother	Infant	Mother	Infant	Mother	Infant	Mother	Infant †	Mother
Age	5 m	18 y	7 m	30 y	4 m	22 y	8 m	25 y	4 m	15 y	5 m	31 y	5 m	18 y	1 d‡	22 y
Image of infant	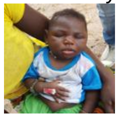		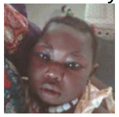		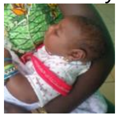		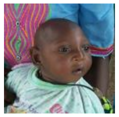		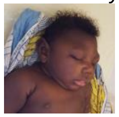		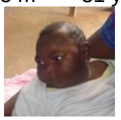		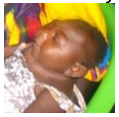		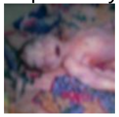	
Region	Bafatá		Bolama		Bafatá		Gabú		Farim		Bafatá		Bafatá		Gabú	
Village	Tendito		Bolama		Cambadju		Dara		Farim		Xitole		Xitole		Gabú	
Symptoms of mother during pregnancy	Fever, myalgia, headache, weakness, stomach pain		Headache, vaginal discharge		Fever, headache, weakness		Fever, arthralgia, myalgia, headache, weakness, anemia (received blood transfusion)		Headache		Weakness, stomach pain		Fever, headache, weakness, stomach pain		None	
Month of birth	May		March		June		February		June		May		May		August	
Birthweight	Not reported		2,600 g		Not reported		Not reported		2,300 g		Not reported		3,200 g		Not reported	
Weight at sampling	Not reported		Not reported		5,800 g		Not reported		Not reported		6,100 g		No info		Not reported	
Infant sex	F		F		F		M		M		F		F		M	
Infant head circumference (*1*) at birth<br>Girls: reference range (31.7–36.1 cm)<br>Boys: reference range (32.1–36.9 cm)	Not reported		Not reported		Not reported		Not reported		Not reported		Not reported		Not reported		Not reported	
Infant head circumference at sampling<br>Girls: reference range (39.0–43.9 cm)<br>Boys: reference range (40.3–44.8 cm)	34 cm		35 cm		33 cm		36.5 cm		34 cm		34 cm		33 cm		Not reported	
Clinical symptoms	Microcephaly, malformed umbilicus		Microcephaly, abnormal psychomotor development		Microcephaly		Microcephaly, conjunctivitis, abnormal psychomotor development		Microcephaly		Microcephaly, visual problems		Microcephaly		Microcephaly	
Pathogen, diagnostic test																
*Z*ika virus																
PCR§	–	–	–	–	–	–	–	–	–	–	–	–	–	–	NA	–
IgM¶	–	–	–	–	–	–	–	–	–	–	–	–	–	–	NA	–
IgG¶	+	+	+	+	+	–	–	+	+	+	+	+	+	+	NA	–
Nab#	>640	640	>640	120	140	560	18	>640	>640	>640	240	>640	50	480	NA	–
Dengue virus																
IgM¶	–	–	–	–	–	–	–	–	–	–	–	–	–	–	NA	–
IgG¶	+	–	–	–	–	–	–	+	+	+	+	+	–	+	NA	–
Nab#	–	NA	NA	–	–	NA	–	NA	-	NA	-	NA	–	NA	NA	NA
Chikungunya virus																
IgM¶	–	–	–	–	–	–	–	–	–	–	–	–	–	–	NA	–
IgG¶	–	+	–	–	–	–	–	+	–	–	–	+	–	–	NA	–

	Case															
Category	H		I		J		K		L		M		N		O	
Patient	Infant	Mother	Infant	Mother	Infant	Mother	Infant	Mother	Infant	Mother	Infant	Mother	Infant	Mother	Infant †	Mother
Toxoplasma gondii																
IgM**		NA		–		–		NA		–		NA		–		–
IgG**		NA		–		–				–		NA		–		–
Treponema pallidum																
IgG††		NA		–		–		NA		–		NA		–		–
Parvovirus																
*q*PCR‡‡	–		–		–		–		–		–		–		NA	
IgG¶¶		NA		–		0.41		NA		1.76		NA		–		INC
Varicella-zoster virus																
*q*PCR‡‡	–		–		–		–		–		–		–		NA	
IgG††		NA		200		200		NA		400		NA		400		800
Rubella virus																
*q*PCR‡‡	–		–		–		–		–		–		–		NA	
IgG§§		NA		131		120		NA		101		NA		>200		178
Cytomegalovirus																
*q*PCR‡‡	36	–	–		31	–	–		–		36	–	34	–	NA	
IgM††	200	–		NA	–	–		NA		NA	–	–	400	200		NA
IgG††	160	NA		160	80	40		NA		320	80	NA	80	40		320
Herpes simplex virus																
*q*PCR‡‡	–	–	–		–		–		–		–		–		NA	
IgG††		NA		2.01		2.03		NA		1.94		NA	–	2.00		2.24

*INC, inconclusive; NA, not analyzed; Nab, neutralizing antibody; RT-qPCR, reverse transcription quantitative PCR; qPCR, quantitative PCR.
†Samples not send to Statens Serum Institut (Copenhagen, Denmark) for analysis.
‡Deceased.
§In-house PCR modified from Faye et al (*2*).
¶ Arbovirus Fever Mosaic 2 (Zika virus, chikungunya virus, dengue virus) (IgG/IgM) (Euroimmun AG,Luebeck, Germany).
#Neutralization assay with African strain MR766. Positive antibody titers are shown.
**VIDAS TOXO IgG II (BioMerieux, Marcy l'Etoile, France). Positive antibody titers are shown.
††In-house ELISA. Positive antibody titers are shown.
‡‡In-house qPCR. Positive Cq values are shown.
§§ Enzygnost anti-Rubella-Virus IgG (Siemens Healthineers, Erlangen, Germany). Positive antibody titers are shown.
¶¶LIAISON Biotrin Parvovirus B19 IgG (DiaSorin, Saluggia, Italy). Positive antibody titers are shown.

References

1. World Health Organization. Child growth standards [cited 2018 Jan 30]. http://www.who.int/childgrowth/standards/hc_for_age/en/

2. Faye O, Faye O, Diallo D, Diallo M, Weidmann M, Sall AA. Quantitative real-time PCR detection of Zika virus and evaluation with field-caught mosquitoes. Virol J. 2013;10:311. PubMed http://dx.doi.org/10.1186/1743-422X-10-311
